# Impact of Interactive Learning Environments on Learning and Cognitive Development of Children With Special Educational Needs: A Literature Review

**DOI:** 10.3389/fpsyg.2021.674033

**Published:** 2021-04-29

**Authors:** Leire Ugalde, Maite Santiago-Garabieta, Beatriz Villarejo-Carballido, Lídia Puigvert

**Affiliations:** ^1^Departament of Educational Organization and Didactics, University of the Basque Country, San Sebastian-Donostia, Spain; ^2^Faculty of Psychology and Education, University of Deusto, Bilbao, Spain; ^3^Department of Sociology, Autonomous University of Barcelona, Barcelona, Spain; ^4^Department of Sociology, University of Barcelona, Barcelona, Spain; ^5^Affiliated Member of the Centre for Community, Gender and Social Justice, Institute of Criminology, University of Cambridge, Cambridge, United Kingdom

**Keywords:** interaction, learning, development, learning environments, special educational needs

## Abstract

Children with special educational needs (SEN) achieve lower educational levels than their peers without special needs, leading to a higher risk of social exclusion in the future. Inclusive education aims to promote learning and to benefit the cognitive development of these students, and numerous research studies have indicated that interactive environments benefit inclusion. However, it is necessary to know how these inclusive environments can positively impact the academic improvement and development of these students' cognitive skills. This article provides a review of the scientific literature from Web of Science, SCOPUS, ERIC, and PsychINFO to understand the impact of interactive environments on the academic learning and cognitive skill development of children with SEN. A total of 17 studies were selected. Those studies showed the effectiveness of interactive learning environments in promoting instrumental learning, increasing academic involvement, and improving the cognitive development of children with disabilities. Based on these results, it can be concluded that interaction-based interventions with an inclusive approach nurture the learning and cognitive development of students with SEN.

## Introduction

People with disabilities are among the most vulnerable groups in society. According to the World Health Organization (2011), students with special educational needs (SEN) achieve lower educational levels than non-disabled students, with lower retention rates and promotion within the educational systems. These low educational levels influence subsequent opportunities, as students with SEN are more likely to suffer high unemployment rates, poverty, and wage discrimination (O'Keefe, 2007; Fuchs, 2014). This scenario worsens in adverse situations such as the current COVID-19 pandemic, in which, as reported by the Report of Progress Toward the Sustainable Development Goals (United Nations, 2020), people with disabilities are affected disproportionately.

This reality must be understood in the context of the individual conditions of students with disabilities or other special needs and the educational provisions they receive. For this reason, the United Nations 2030 agenda aims to ensure inclusive and equitable education and promote lifelong learning opportunities for all. The concept of inclusive education has changed from being understood throughout history as a concept that emphasizes the importance of educating students with SEN in conventional classes to transforming schools to facilitate the acquisition of relevant learning by diverse students and to promote belonging to the group (Ainscow, 2005; Meijer, 2010; Porter, 2011; Hansen et al., 2020). Thus, inclusion is an initiative that leads to the improvement of educational systems and the promotion of more equitable societies (Arnesen et al., 2007; Graham and Slee, 2008; Vlachou et al., 2016).

However, inclusive education is one of the most important challenges facing schools today, especially for SEN students. The latest data available for Europe (European Agency for Special Needs Inclusive Education, 2020) show that the percentage of students in primary and lower secondary education with an official SEN decision who follow education in mainstream classes alongside their mainstream peers at least 80% of the school time is 64.97% (data from 29 countries). Although the percentage of students with SEN enrolled in mainstream schools is quite high and the European Agency reports a slight increase in students with SEN placed in mainstream schools, the same agency warns that this does not mean that these students are integrated into the mainstream classroom with the rest of the students. The European Agency also refers to the trend in all the countries studied of still placing the students with the most severe SEN in special education schools (European Agency for Special Needs Inclusive Education, 2018). The use of special education or support classrooms has traditionally been linked to the concept that the particular needs of students with SEN are best met in specially designed environments adapted to their abilities (Etscheidt, 2006). However, studies in education have shown that the segregation of groups of students, including students with SEN, decreases their opportunities for learning and interaction with society (Fitch, 2002; Bossaert et al., 2015). Separated education also causes negative consequences such as low expectations regarding one's own abilities and decreased self-confidence, academic performance, and self-esteem (Fisher et al., 2002; Fitch, 2002; Stepaniuk, 2019).

Conversely, several investigations have shown that the integration of students with SEN in conventional classes and schools is associated with positive effects on social and cognitive development (Peetsma and Van der Veen, 2015). Regarding academic learning, Dessemontet et al. (2012) conducted a comparative study of children with intellectual disabilities who attended a general education classroom or special schools and found better literacy skills in the first group. The same type of comparison was made by Laws et al. (2000) with children who had Down Syndrome, and in this case, those who participated in the mainstream setting achieved better learning results, including higher scores for vocabulary, grammar, and digit span measures.

The opportunities for interaction and dialogue with typically developing peers may play a role in obtaining positive achievements regarding learning promotion in mainstream contexts, which contribute to reducing inequalities and enhancing inclusion. The importance of dialogue and interaction in the development and learning of children with and without SEN were already stressed by Vygotsky (1978). Similarly, social and dialogical interactions are identified as an important contributing factor for language acquisition (Purcell-Gates et al., 2011), scientific reasoning (Howe, 2009), and mathematical understanding (Stein et al., 2015). For this reason, to promote an improvement in learning, it is important to consider the creation of dialogical learning environments in which classroom interactions and dialogues include all students (Berry and Englert, 2005; Ni Bhroin, 2013). Research such as that carried out by Berry and Englert (2005) and Rajala et al. (2012) shows the improvements produced in students' development and learning as a result of the increase in opportunities for students with and without SEN to participate more actively in classroom dynamics. Within the efforts to advance toward more inclusive education where learning interactions and dialogues are promoted among diverse students, schools as learning communities implement successful educational actions (SEAs) (Flecha, 2015) with students grouped according to heterogeneity criteria, avoiding any type of segregation and enhancing the richness of interactions (Díez-Palomar et al., 2020). Several studies have reiterated the effectiveness of SEAs in the creation of inclusive learning contexts, which benefit students with SEN (García-Carrión et al., 2018). In these investigations, quality interactions among diverse students have been found to be a relevant factor for achieving positive impacts.

Based on this existing knowledge, there is a need to further explore the potential of interactive learning environments to create enhanced opportunities for students with SEN concerning their academic learning and cognitive skills development. With the aim of delving deeper into the aspects that can help optimize the learning processes of pupils with SEN, this study aims to identify and systematize the existing contributions published in recent scientific literature on the impact of educational interventions based on dialogue and/or interaction on the academic improvement and development of children with SEN.

## Materials and Methods

To conduct the systematic review presented in this study, the PRISMA (Moher et al., 2009) recommendations were taken into account. In this way, the systematic research of the literature was conducted based on the main databases in the fields of Psychology and Education: Web of Science (WoS), SCOPUS, ERIC, and PsychINFO. Search terms were selected based on four categories: effects, target, intervention, and population/context. Taking into account the research goal and the most common terms used in education in these fields, the following keywords per category were selected: (a) effects: “inclusion,” “cognitive development,” and “skills”; (b) target: “disabilities,” “special needs,” “special educational needs,” and “teachers”; (c) intervention: “interaction,” “interactive learning environment,” “interactive learning,” “dialogue,” “dialogic interaction,” and “dialogic teaching and learning”; and (d) population/context : “children,” “student,” “classroom,” “school,” and “pupil.” The literature published between 2005 and 2020 was searched, ensuring a broad and updated review of the published evidence on the subject.

The final search equation was defined using the Boolean connector “AND,” and combinations of the keywords were made by securing a keyword for each of the four search categories. The search was filtered by scientific documents and by the area of knowledge of social science in WoS. A total of 544 searches were carried out, and 3,697 articles were identified.

### Inclusion Criteria and Exclusion Criteria

The selection of the articles was carried out using the following inclusion criteria: (i) educational intervention for students with SEN in school settings, (ii) educational intervention based on interaction/dialogue with students with SEN in school settings, and (iii) evidence of improvement in learning and development (reading, attention, language, oral expression, reasoning, curricular content) and cognitive development. The criteria for exclusion were as follows: (i) 18 years of age and older, (ii) duplicate citations, (iii) out-of-school interventions, and (iv) interventions not related to disabilities/special educational needs.

**Figure 1:**
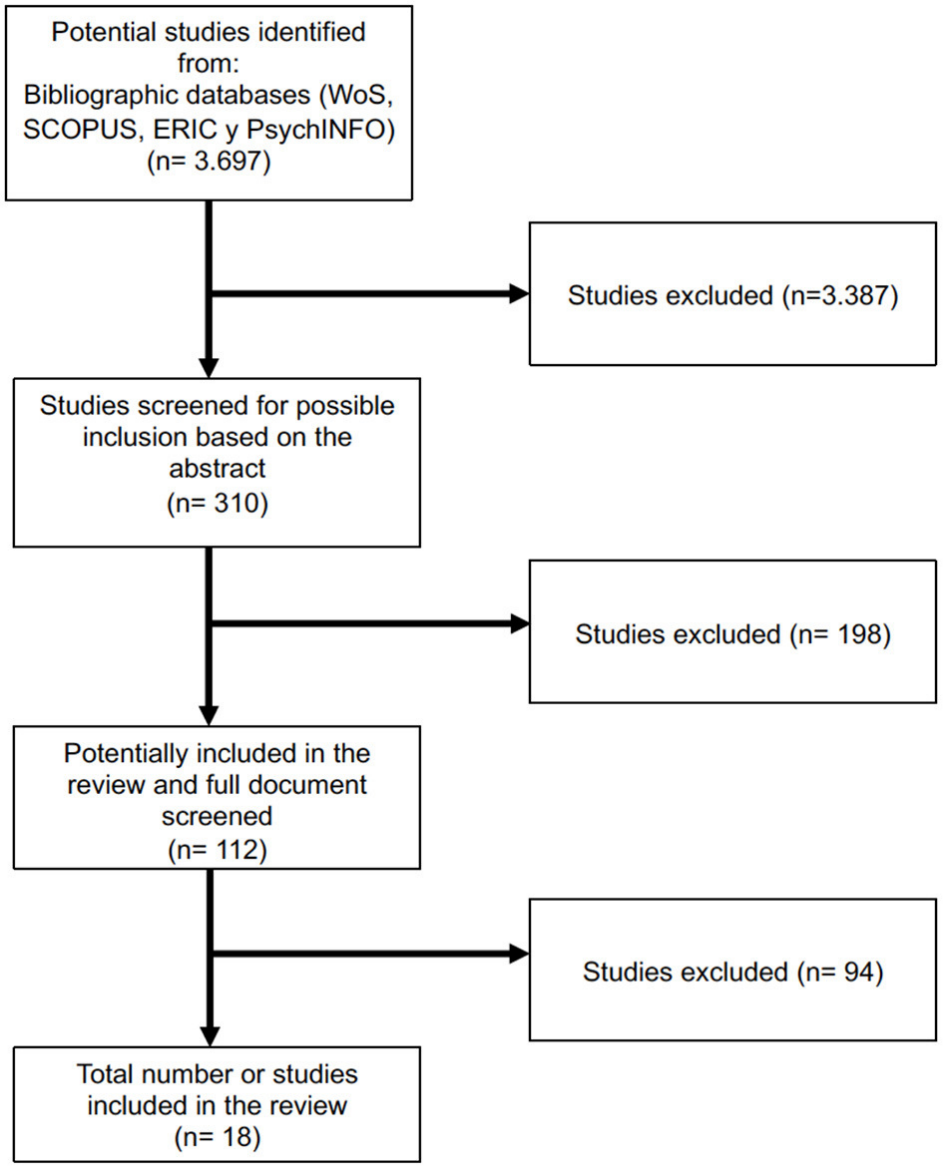
Flow diagram to show the process of study selection.

Articles that met all the inclusion criteria in their abstracts were preselected for further in-depth reading of the entire article. Articles that met at least one of the exclusion criteria were not selected. A total of 310 papers were preselected based on the abstract, of which 112 articles were selected for downloading and in-depth reading (Figure 1).

## Results

The final selection included 17 scientific articles that provided evidence regarding the academic and developmental impact of interactive learning environments on students with SEN. Table 1 shows a summary of the information on each of the articles organized by the impact generated on the child and indicating the country where the study was conducted, the sample of participating students, the design of the research, the educational program studied, and the main findings of the study.

**Table 1:** Summary of articles from the literature review.

**References**	**Country**	**Sample (Age)**	**Research design**	**Educational program**	**Key findings**
**Impact on literacy learning, language development and communication skills**					
Chen et al. (2020)	USA	448 children, 178 had identified disabilities	Quantitative	Peers' Language Resources	Peer language resources were influential in promoting students' language skills.
Ferguson et al. (2020)	USA	53 children with ASD (3–5 years)	Quantitative	Characteristic of Early Intervention Program	Inclusive early intervention placements encourage to talk more and receive more verbal information from their peers.
García-Carrión et al. (2016)	Spain	9 units of early childhood and primary education	Qualitative	Interactive Groups and Dialogic Literary Gatherings	Increase in written expression, self-confidence in the reading and writing process, progress in reading.
Nahmias et al. (2014)	USA	98 preschools children with ASD	Quantitative	Early Intervention Program	Increased cognitive development of children with SEN.
Parker and Kamps (2011)	USA	2 students with AD	Quantitative	Summer School Program	Increase in vocabulary, interest in literacy activities, skills, and confidence.
Raver et al. (2014)	USA	4 children with hearing loss	Quantitative	Oral Preschool and Inclusive Preschools	Increase in verbal comments and play turns in interventions. Improvement in behaviors with both interventions.
Stanton-Chapman et al. (2008)	USA	120 preschool children at high risk for language and social problems	Quantitative	Head Start	Increased vocabulary and increased frequency of verbal behavior.
Tobin (2007)	USA	4 students with mild intellectual disabilities and 1 student with a learning disability	Qualitative	Positive Interactions and “Good New Visits”	Enhanced literacy learning and made text more accessible.
Whalon and Hart (2011)	–	Children with ASD will - school and post-school opportunities.	Qualitative	Reading Comprehension	Encourages the occurrence of new, spontaneous initiations, and responses during reading.
**Impact on science learning and mathematical thinking**					
Lei et al. (2020)	USA	1 Learner and 1 native english with disabilities	Quantitative and qualitative	PGBM-COMPS Math	Increases the dually classified students' capacity to think and answer multiplicative problems.
Lambert et al. (2020)	USA	A fifth-grade student with autism disorder	Qualitative	Mathematic	Increases verbal and non-verbal participation and mathematical thinking in multiple contexts.
McLure (2020)	Australia	3 students with SEN	Quantitative and qualitative	Thinking Frames Approach	Growth in self-efficacy perception, performance in the evaluation's tasks, engagement and science conceptual understanding.
Wu et al. (2020)	Taiwan	3 students with disabilities (8–9 years)	Quantitative	Peer Mediated Instruction with Augmentative and Alternative Communication and Speech Generating Devices	Improved participants' science knowledge.
**Impact on enhancing academic engagement**					
Andzik et al. (2016)	USA	23 students (6–11 years)	Quantitative	Augmentative and Alternative Communication systems	Increasing the expectations for communication participation and purposefully creating high-quality and diverse interaction opportunities.
Bock (2007)	USA	1 student with AS in a Middle School	Quantitative	Social–behavioral Learning Strategy Intervention	Increasing the percentage of time spent learning and the participant presented long-term memory.
Carter et al. (2015)	USA	21 High Schools	Qualitative	A practical and promising approach for supporting students	Promoting academic learning
Carter et al. (2017)	USA	4 students with ASD of four High School	Quantitative	General Education Classrooms	Improved attitudes, personal growth and a stronger commitment to inclusion.

The studies reviewed show that interactive learning environments improve cognitive skills and the development of instrumental learning in pupils with SEN. Overall, the development of language and literacy competencies and mathematical and science knowledge is highlighted. These studies show that interactions with other students, as well as among students and teachers, in the educational context have a key role in achieving such improvements. In this regard, drawing on the analysis of the 17 studies, the main results concerning the impact generated by interactive learning environments on students with SEN have been organized into three main topics: (1) impact on literacy learning, language development and communication skills, (2) impact on science learning and mathematical thinking, and (3) impact on enhancing academic engagement.

### Impact on Literacy Learning, Language Development, and Communication Skills

The impact of interactive environments on improving language, literacy and communication skills in children with SEN is the most prominent in the studies reviewed. Of the 17 articles selected, a total of nine articles have shown evidence in this regard. In terms of language learning, studies such as that conducted by Chen et al. (2020) in preschool classrooms highlight the significant impact of language resources provided by peers, especially for students with disabilities. When these language resources are shared, there is a considerable language growth effect on students in this sample. The work developed by Ferguson et al. (2020), which focused on preschool students diagnosed with autism, points in the same direction. According to the results of their study, these students received greater verbal input, produced greater verbal output and had access to similar levels of teacher talk when they were integrated in inclusive classrooms compared with those who were in classes with only autist peers or in classes with peers with diverse disabilities. Indeed, being in inclusive classrooms broadens the opportunities for students with SEN to get exposure to natural language in a social context.

One of the key elements in improving children's literacy learning with disabilities is that the interactions promoted are of high quality, mediated by appropriate training and guidance on specific strategies for a specific purpose. Tobin (2007), who studied positive interactions within inclusion experiences, noted that high-quality discussions improved literacy learning and made the text more accessible to children with intellectual and learning disabilities. In this regard, research shows that the interactions established between students with SEN and the rest of the educational community are essential to enhance their learning. The study by García-Carrión et al. (2016) analyzed the impact of interactions between the students themselves and with teachers and adult volunteers (family members, community members, and university students) in the Dialogic Literary Gatherings (DLG) and the Interactive Groups (IG). These are based on an inclusive educational approach where the needs of diverse learners are addressed in a common framework and learning content and activities are shared with the rest of the group. The results revealed that these educational actions contributed to supporting learning, helped students with SEN understand concrete activities, created new learning opportunities, and helped develop new academic skills. Pupils with severe difficulties in written expression increased their self-confidence in completing the writing of a text with coherence.

In the same vein, Parker and Kamps (2011) analyzed written tasks with self-monitoring to teach functional skills and verbal interactions to two students with autism in social settings with peers. The researchers found positive effects on developing learning skills in children with SEN and observed that pupils had improved basic skills (language, mathematics, environmental awareness, autonomy, and social skills). These results are consistent with those obtained by Stanton-Chapman et al. (2008) in their study on the effects of a multicomponent intervention strategy to increase peer-directed social communication in eight children at risk of poor language and social skills development. The results of this study indicated that the children had increased vocabulary, frequency of verbal behavior, and social competence, especially in establishing friendships.

Research especially highlights the impact of learning interactions on language and communication for students with autism spectrum disorder (ASD). Nahmias et al. (2014) examined the association between cognitive outcomes and the receipt of early intervention for students with ASD in three settings: with other students with autism, with students with various disabilities, and in inclusive settings. The main finding was that children in inclusive settings experienced greater average gains in cognitive scores, especially in language and social communication, than did some children who attended classrooms without typically developing peers. These results were most visible in children with more severe social disabilities, with lower adaptive behavior skills, and in those with at least some form of expressive or receptive communication. Children with more severe social disorders in inclusive settings benefited from the more sophisticated social, emotional, and adaptive strategies displayed by their peers. Thus, Nahmias et al. (2014) noted that inclusive schooling for children with ASD increased opportunities to interact with and learn from typically developing peers, which may be significant for their cognitive development. The authors noted that mixed disability placement could be inadequate for children with autism spectrum disorders because it provides the fewest opportunities to either interact with typically developing peers or receive an autism-specific intervention.

Additionally, focusing on students with autism, Whalon and Hart (2011) analyzed the possibilities for adapting an evidence-based program to develop their reading skills. The selected intervention consisted of question-and-answer relationships (QARs), a generative questioning strategy used to promote reading comprehension in typically developing students. The study identified a way to adapt this strategy to include instructional supports that (a) provide a way for students with ASD to attend to important elements of the text immediately; (b) successfully engage in reciprocal interactions about the text; and (c) encourage new initiations and spontaneous responses during reading. By creating opportunities for students with ASD to interact with their peers through an activity based on direct and explicit reading comprehension, students with ASD are encouraged to learn a strategy that not only helps them access the general education reading curriculum but also provides them with the tools to engage in meaningful academic discussions with their peers, thus furthering their social and educational communication goals.

Similarly, Raver et al. (2014), in their study of children with profound hearing loss, found that the majority of these children benefited from structured opportunities of interaction with typically hearing children to learn verbal skills, and both groups improved behavior in prelinguistic interventions.

### Impact on Science Learning and Mathematical Thinking

As shown in the previously mentioned studies, a relevant aspect to improve the quality of learning for students with SEN in inclusive settings is to identify what specific supports can help them participate and interact effectively in learning activities. This is relevant for language-related learning and mathematics and science learning. Lei et al. (2020) studied the case of a fifth-grade student dually classified as English Learner (English would be her second language) and Learning Disabilities to analyze the types of educational scaffolds that mathematics teachers can use to support multiplicative reasoning effectively. Four types of teacher scaffolding (visual, linguistic, interactive and kinesthetic) were studied during seven sessions of mathematical instruction. In turn, three different interaction contexts were considered for the interactive scaffolding: (1) teacher-student interaction, (2) student-student interaction, and (3) small group interaction. Small-group interaction was the most effective interaction context, as the student showed an increased ability to think and respond to multiplicative problems in small group contexts. Moreover, kinesthetic and linguistic scaffolds were found to be the most beneficial in helping the student cultivate mathematical thinking, with both concrete and abstract units. These types of scaffolds also contributed to generating more elaborate language use of mathematical content.

Another study that demonstrates the impact of interactive educational contexts in improving the learning of children with ASD is that carried out by Lambert et al. (2020) with a fifth-grade student with autism. The authors demonstrated how, thanks to an intervention in the classroom in which the participation rules were made more explicit and additional scaffolds (such as greater responsibility of the peers and more collaborative actions) were incorporated, the child became able to explain his mathematical thinking in multiple contexts. Similar improvements were observed by Wu et al. (2020) when analyzing the impact of a peer-mediated intervention (PMI) on the learning of science by students with cognitive disabilities. Nine non-disabled peers taught scientific concepts to their disabled peers through questions about the content and modeling and encouraged their peers to use the iPad-SGD. The results showed that peer participation, aided by augmentative and alternative communication (AAC) and using speech-generating devices (SGDs), managed to improve the communication of the target participants with their peers during the scientific experiments and improved the specific scientific knowledge of the participants.

Finally, the case study published by McLure (2020) presents the experience of a student with severe special educational needs in accessing science learning with the thinking frames approach (TFA), in which students are organized into heterogeneous groups to predict the outcome of carefully designed problems. To do so, they discuss with their peers their conceptions and contrast them with those of the others, generating a social construction of knowledge. The results of the study revealed improvements in various aspects for all students, which were possible due to the interactions established for the collaborative elaboration of productions. Especially for students with SEN, because of peer interactions and support, students experienced improvements in participation in small and whole groups, perception of self-efficacy and classroom assessment activities.

### Impact on Enhancing Academic Engagement

The studies analyzed also report results of peer support and other focused interventions in terms of engagement in academic learning and interactive learning situations. In this regard, the researchers noted that social interactions in learning contexts could create additional communication opportunities for promoting inclusion and learning for students with disabilities, nurturing other social behaviors, and raising engagement in educational activities (Carter et al., 2015, 2017; Andzik et al., 2016).

One of these studies (Bock, 2007) examined the effect of a social-behavioral learning strategy intervention (Stop-Observe-Deliberate-Deliver-Act; SODA) on the interaction skills for engaging in cooperative learning activities, playing board games, and visiting peers during lunch of a high school student with Asperger syndrome (AS). The child participated in cooperative learning activities with peers in a cooperative learning group. The study found that the participant had a higher percentage of time spent learning cooperatively, playing board games, and visiting during lunch when he began SODA training. Additionally, the effects were maintained after the intervention.

Another study (Carter et al., 2017) examined the impact and social validity of peer support-based student arrangements with four high school students with autism spectrum disorder (ASD), looking at social interactions with peers and academic engagement. The researchers used momentary time sampling to measure academic engagement to document whether the student was consistently engaged, inconsistently engaged, or disengaged. The overall results indicated that all four students increased social interactions with their peers, while academic engagement increased or was maintained for three of the students. According to the authors, these results suggest that a greater emphasis on the design and delivery of academic support is needed to further improve learning outcomes. In light of the results, peer support strategies should be considered for this purpose.

## Discussion

The literature review carried out finds that interactive learning environments have a positive impact on improving academic learning and cognitive skills development in children with SEN. Although further research is needed on this aspect, the 17 selected studies shed light on the importance of implementing interaction-based learning environments. Their benefits have been evidenced for developing language, literacy, and communication skills for SEN pupils (Whalon and Hart, 2011, among others; Chen et al., 2020; Ferguson et al., 2020), for the acquisition of mathematical competence and science learning (Lambert et al., 2020; Lei et al., 2020; Wu et al., 2020) and for enhancing engagement in learning (Bock, 2007; Carter et al., 2017).

One of the aspects in the reviewed studies is the key relevance of the interaction between peers when it allows students to support each other and creates opportunities for learning from each other collaboratively. This is relevant because, as Gee et al. (2020) emphasize, learners with SEN tend to reduce the extent of their interactions when they are in segregated settings. However, the opposite occurs in inclusive environments, where interactions increase. In this regard, research shows that it is necessary not only to allow students with and without special needs to interact but also to provide peers who accompany students with functional diversity with tools so that they can manage interactive situations and provide the necessary support (Carter et al., 2017). This would empower students with disabilities to communicate effectively with peers and provide peers with tools to help their classmates, which are both vital factors for the cognitive and learning development of students with SEN. This is consistent with other studies that reinforce the idea that when teachers promote educational actions that increase student interactions oriented to learning, they can increase levels of instrumental learning (Ni Bhroin, 2013), including language learning (Purcell-Gates et al., 2011) or mathematical skills (Stein et al., 2015). Importantly, our review of research also found that benefits often do not appear separately, but improvements in communication, literacy, scientific or mathematical learning and engagement in learning situations can occur simultaneously as a result of participating in interactive learning environments. In addition, the benefits reported are not limited to specific disabilities or special needs; on the contrary, the studies reviewed covered a wide range of learning difficulties (related to autism, hearing loss, intellectual disabilities, learning disabilities, and other special needs) and, more importantly, we found that the impact of the interactive learning situations helped students' progress in the areas that were precisely more affected due to their disability (such as communication in the case of students with autism and hearing loss or literacy in the case of students with a learning disability). This indicates that interactive learning environments can contribute to reducing the impact of students' disabilities on their learning and development.

Furthermore, research shows that classroom interactions are positive not only when they occur between people directly involved in the school, such as teachers and pupils, but also when they involve other people from the community, as shown in the study by García-Carrión et al. (2016) on the impact of the Dialogic Literary Gatherings and the Interactive Groups to enhancing the learning and expectations of students with SEN. In this regard, it is also important to note that interactive learning environments that are effective with students with SEN, such as DLG, are also effective for the rest of the students, contributing to the emergence of school-relevant language and literacy for all students (Lopez de Aguileta, 2019). Boyle et al. (2019) also pointed out the benefits of shared reading, not only with teachers but also with parents, in improving literacy skills in children with ASD. These results are congruent with the indications gathered in The Information and Communication Technology for inclusion report (European Agency for Special Needs Inclusive Education, 2013), in which it is indicated that schools need to involve a greater diversity of agents, creating formal and informal networks that support their practice and working as communities of practice. Within these communities, all those individuals or organizations that share a common interest participate, including families, which can be involved in the development of proposals for students. In these communities, ideas and ways of working can be exchanged, which help identify problems and solutions. Families must be part of these communities and be involved in the development of proposals for students. In this way, the report is committed to creating working models that involve students, teachers, parents, and other professionals working together to educate all students.

According to the European Agency for Special Needs Inclusive Education (2011), students' active participation is one key element to achieve the objective of implementing inclusive education for all. The conclusions of this research review contribute to this aim by showing how contexts of interactive learning can increase these students' participation in shared learning settings while enhancing their learning and cognitive development.

However, we cannot ignore the limitations of this study. As can be seen in Table 1, the vast majority of the collected research has been developed in the United States. Future research should focus its efforts on broadening this topic's study contexts, analyzing the effects of interactive learning context on students with SEN in other countries. In this regard, it should also be taken into account that the majority of articles in the platforms on which the searches have been carried out are written in English, which raises the question of whether there may be studies conducted in other countries and published in other languages and journals that are not included in the databases used in this study. Finally, it should be taken into account that the concept of interaction is broad so that the articles collected gather evidence referring to different types of interaction and with different types of special needs. It would be interesting if future research could continue to investigate the ideal characteristics that the different contexts and agents involved in these interactions should meet to obtain the best learning outcomes for students with SEN and if the research samples could represent the greatest possible diversity of these students.

## Data Availability Statement

The original contributions presented in the study are included in the article/supplementary material, further inquiries can be directed to the corresponding author/s.

## Author Contributions

LP: conceptualization. MS-G, LU, and BV-C: methodology, formal analysis, and writing—review and editing. MS-G: data curation. BV-C: writing—original draft preparation. All authors have read and agreed to the published version of the manuscript.

## Conflict of Interest

The authors declare that the research was conducted in the absence of any commercial or financial relationships that could be construed as a potential conflict of interest.
